# Cancer Risk in the Heart Failure Population: Epidemiology, Mechanisms, and Clinical Implications

**DOI:** 10.1007/s11912-020-00990-z

**Published:** 2020-12-02

**Authors:** Alessandra Cuomo, Flora Pirozzi, Umberto Attanasio, Riccardo Franco, Francesco Elia, Eliana De Rosa, Michele Russo, Alessandra Ghigo, Pietro Ameri, Carlo Gabriele Tocchetti, Valentina Mercurio

**Affiliations:** 1 Department of Translational Medical Sciences, Federico II University, Naples, Italy; 2 Department of Molecular Biotechnology and Health Sciences, Molecular Biotechnology Center, University of Torino, Torino, Italy; 3 Cardiovascular Disease Unit, IRCCS Italian Cardiovascular Network, IRCCS Ospedale Policlinico San Martino, Genoa, Italy; 4 Department of Internal Medicine, University of Genova, Genoa, Italy; 5 Interdepartmental Center of Clinical and Translational Research, Federico II University, Naples, Italy

**Keywords:** Cardio-oncology, Cancer, Heart failure, Aging, Pathophysiology, Risk factors

## Abstract

**Purpose of Review:**

Along with population aging, the incidence of both heart failure (HF) and cancer is increasing. However, little is known about new-onset cancer in HF patients. This review aims at showing recent discoveries concerning this subset of patients.

**Recent Findings:**

Not only cancer and HF share similar risk factors but also HF itself can stimulate cancer development. Some cytokines produced by the failing heart induce mild inflammation promoting carcinogenesis, as it has been recently suggested by an experimental model of HF in mice.

**Summary:**

The incidence of new-onset cancer is higher in HF patients compared to the general population, and it significantly worsens their prognosis. Moreover, the management of HF patients developing new-onset cancer is challenging, especially due to the limited therapeutic options for patients affected by both cancer and HF and the higher risk of cardiotoxicity from anticancer drugs.

## Introduction

Cardiovascular disease and cancer are among the leading causes of death worldwide and the first two causes of death in the industrialized countries [1, 2]. As the world population grows older, the incidence of both cancer and cardiovascular diseases increases, and it is expected to keep on growing in the next decade [3]. Heart failure (HF) is the main complication of several cardiovascular diseases, and its incidence has grown enormously over the past decades [4••, 5]. It is also well known that cancer patients may develop HF due to cardiotoxic effects of antineoplastic treatments [6••, 7••, 8]. In order to provide better cardiological care to oncology patients, the field of cardio-oncology is acquiring an increasingly relevant role [9, 10]. An important challenge in cardio-oncology is played by the onset of cancer in patients with preexisting HF [11]. While HF and cardiotoxicity induced by antineoplastic treatments have been largely studied over the years, there is scant data about cancer in preexisting HF patients. Moreover, patients with cardiac dysfunction are usually excluded from oncological clinical trials, due to the high prevalence of comorbidities and to their worse prognosis, compared to general population [1].

**Fig. 1:**
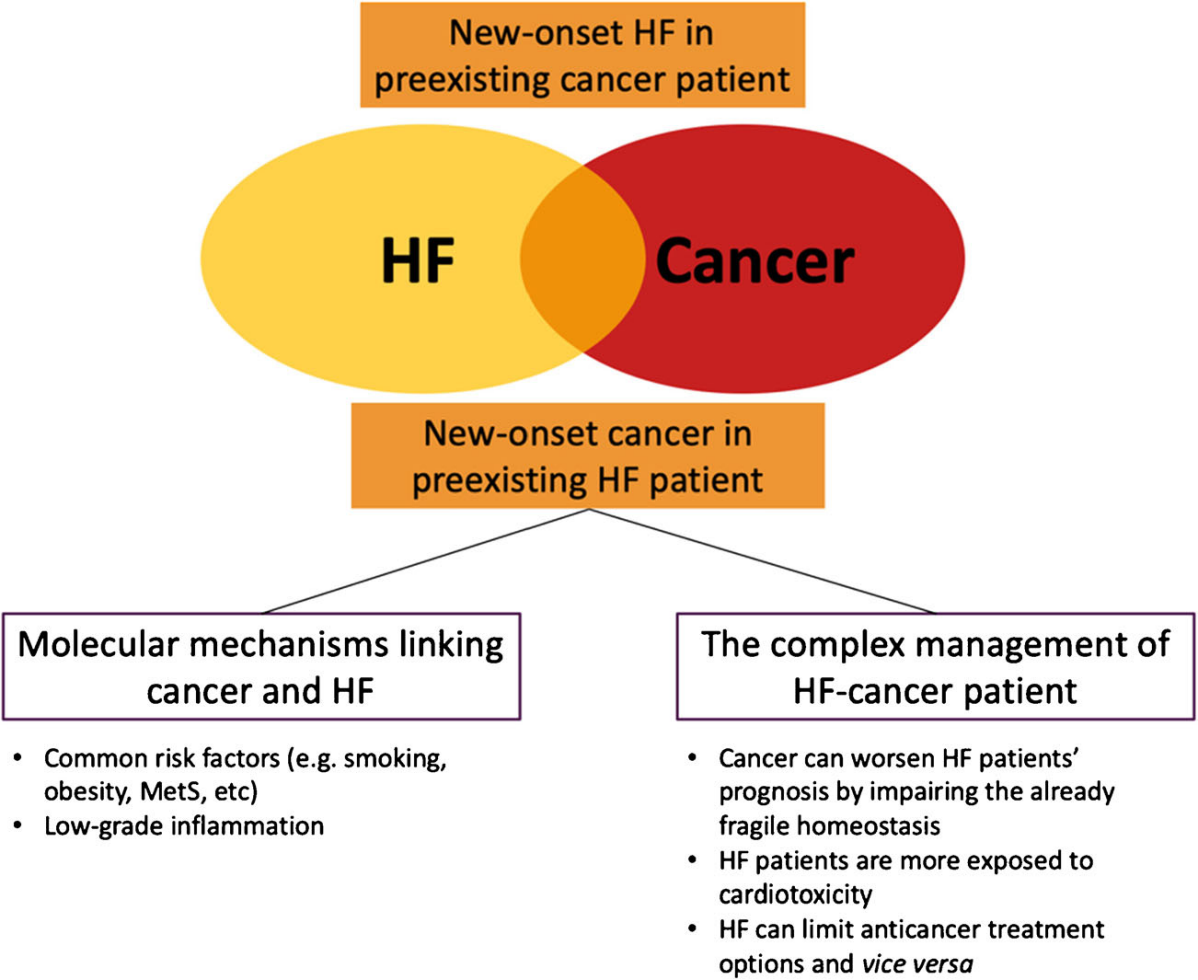
Graphic summary of the mechanisms and the clinical implications of the interaction between heart failure and cancer

Aim of this review is to discuss epidemiologic evidence concerning cancer incidence in HF patients and the most recent findings on the possible mechanisms underlying the development of cancer in the setting of HF, and to highlight challenging clinical implications. Figure 1 summarizes what we discuss in this review.

### Cancer in the Heart Failure Population: Epidemiology

The incidences of both cancer and HF have increased in the past decades, and the risk of developing one of these two diseases augments over the lifespan [5]. Moreover, some studies have explored the incidence of cancer in patients with prior diagnosis of HF, and it has been demonstrated that there is indeed an increased risk [12•, 13•]. Hasin and colleagues explored the risk of cancer incidence in patients with HF in a case-control study (the study included 596 patients with HF and 596 controls), showing that patients with HF had a 60% higher risk of developing malignancies, compared to non-HF controls. These results were confirmed when adjusting for some of the most common risk factors of both cancer and HF, such as BMI, smoking, and diabetes [14]. Gastrointestinal and male reproductive systems were the most incident neoplasms in the cohort. In a prospective cohort study, the same group then further explored the risk of cancer among a homogenous group of myocardial infarction (MI) survivors. The results of this study confirmed an increased risk of cancer among patients who developed HF after MI [15•]. Interestingly, beside cardiovascular mortality, stress echo (SE) has also been shown to predict cancer mortality, acting as a proxy of the shared risk factor milieu for cancer and cardiovascular (CV) death [16]. In particular, in the study from Carpeggiani and colleagues [16], cardiovascular mortality, cancer mortality, and non-cardiovascular and non-cancer mortality were the primary endpoints, and SE was defined positive when at least 2 segments of the same vascular territory showed regional wall abnormalities. The peak wall motion score index was able to predict cardiovascular, cancer, and non-cardiovascular and non-cancer mortality. The authors speculate that the ability of SE in predicting cancer deaths could be linked to the common risk factors and pathophysiology shared between cancer and cardiovascular diseases [16].

In another manuscript, Banke and colleagues explored the incidence of cancer in a Danish group of 9307 patients affected from HF (predominantly with left ventricular ejection fraction < 45%) and demonstrated a higher incidence rate of malignancies compared to the background population (188.9 vs 63.0 with 95% confidence interval), with an incidence rate ratio of 1.24 for cancer [13•]. Specifically, they found that the two most common new-onset malignancies in the examined HF population were the lung and skin, followed by both renal and urinary cancers (which had the same hazard ratio). Basically, all types of cancer were more frequent but prostate carcinoma [13•].

In addition, other important epidemiological data comes from a recent assessment on the prevention of renal and vascular end-stage disease (PREVEND) study population [17], in which, during a median follow-up of 11.5 years, 1132 subjects out of 8319 (13.1%) were diagnosed with cancer. New-onset cancer cases were provided through the nationwide network and registry of histopathology and cytopathology data and archive in the Dutch PALGA [18]. Kaplan–Meier analysis according to NT-proBNP levels showed an increased risk of developing all-cause cancer, even when adjusted for common risk factors between HF and cancer [18].

When considering these data, it should be taken into account that there may be a surveillance bias, due to the fact that these study patients usually undergo an intense follow-up program that may lead to anticipate cancer diagnosis, sometimes discovering malignancies that would have gone undiscovered. Moreover, some of the most common therapies used to treat HF patients may play a role in revealing tumors otherwise asymptomatic (e.g., a latent intestinal neoplasm can induce bleeding due to anti-thrombotic therapy) [1]. Clinical presentations can also be difficult to distinguish between HF and cancer, since the 2 conditions share common symptoms (such as fatigue, dyspnea, weight loss, muscle wasting, edema) [1, 2]. This may delay the diagnosis of new-onset cancer in HF patients due to the overlap in clinical manifestation. Furthermore, CV function and predictors of exercise capacity have been shown to be impaired in patients with cancer per se [19]. Hence, symptoms due to a tumor may overlap with those of HF and be attributed to heart disease. This may even delay cancer diagnosis, as symptoms might be thought of as due to advancing disease rather than new cancer [1].

### Mechanisms of Cancer Development in Heart Failure

Important pathophysiologic mechanisms connect cancer and HF and may explain the higher incidence of cancer in HF patients compared to the general population. First, there are some common risk factors between these two diseases, such as aging, smoking, and metabolic syndrome (MetS) with its components, including obesity and insulin resistance [20]. Moreover, some of the most common mechanisms involved in HF may also have a role in neoplasm growth and dissemination. Finally, another factor to take into account is the low-grade inflammation state typical of HF patients which may constitute a substrate for the development of cancer in these patients [21].

In particular, the cumulative risk of developing chronic diseases increases with aging, with involvement of degenerative mechanisms such as oxidative stress and cellular senescence, which can contribute to the development of cardiovascular diseases and cancer [22–24, 25•]. Additionally, the link between the use of tobacco and malignancies is well known and is globally considered one of the main causes of cancer-related deaths. It has been estimated that approximately 25% of all cancers in men and 4% in women might be connected to smoking, and in both sexes, approximately 16% of cancers in more-developed countries and 10% in less-developed countries may be linked to tobacco use [26], which is also the most important risk factor in developing cardiovascular diseases and an independent risk factor for coronary artery disease [5].

MetS, defined as the presence of at least three conditions among central obesity, high blood pressure, dyslipidemia, and increased fasting glucose, is notoriously considered an important risk factor for cardiovascular diseases [27]. In addition, some findings demonstrate an association between both hyperglycemia [28] and dyslipidemia [29] and increased risk of developing certain types of tumor, while the presence of obesity not only increases the relative risk of developing cancer [30] but also is associated with a higher rate of recrudescence or recurrence [31] and poorer prognosis [32].

Beside common risk factors, other pathophysiological mechanisms are shared by both cancer and HF. Hyperactivation of the sympathetic nervous system (SNS), the renin–angiotensin–aldosterone system (RAAS), and the natriuretic peptide system is among the hallmarks of HF and has been conjectured to possibly increase the risk of cancer [33–35]. When studying the SNS, great attention has been placed to the β-adrenergic receptor (βAR) regulation and activation, hypothesizing that an excess of their activity may lead to tumorigenesis through the β-arrestin-1 signaling [36]; induce cell proliferation by means of molecular pathways such as CREB, NF-kB, and AP-1 [37]; and confer resistance to apoptosis via multiple mechanisms, i.e., inhibition of suppressor gene p53 [37], proapoptotic protein BAD [38], and anoikis [39]. Interestingly, beside playing a fundamental role in heart function, both β1 and β2 ARs are expressed in all cell cancer types and mediate cancer proliferation, hence β-blockers might be putative adjuvants for cancer treatment [40, 41], not only in cardioprotection from anticancer drug–induced cardiotoxicity [42•, 43, 44•].

The SNS may also play a role in modifying the microenvironment in which cancer develops and grows [45], promoting neoplastic dissemination via tumor-associated macrophages [46] that can enhance peri- and intratumoral lymph and blood vessel density by releasing prostaglandin E2 and stimulating VEGF-C expression in response to βAR stimulation [47]. Finally, another effect that SNS might have on cancer natural history is suppression of natural killer cells, through βAR activity, resulting in an easier development and dissemination of neoplasms [48].

RAAS activation has been shown to strongly associate with an increase of tumor angiogenesis and angiogenetic factor expression, invasiveness, metastasis, and overall poor prognosis [49]. Nevertheless, clinically addressing this interaction using RAAS antagonist led to conflicting results in the past [50, 51], even if a large meta-analysis showed a beneficial effect of these therapies on all cancer endpoints [52•]. Indeed, a positive correlation between angiotensin receptor blockers (ARBs) or ACE inhibitors (ACEi) and risk of cancer has been hypothesized, on the basis of some data from the SOLVD and the CHARM studies. The problem in interpretation of these data is the competing risk issue: if HF patients receive life-saving therapies, development of malignancies has more “opportunity” and patients may paradoxically be diagnosed with cancer more frequently [1]. On the other hand, it has been observed that angiotensin II, via its receptor, has a main role in VEGF-dependent angiogenesis and crucial steps in carcinogenesis, including cell migration and proliferation. Hence, angiotensin inhibitors may be potential therapeutics for metastatic renal cell carcinoma [53] or could be added to neoadjuvant therapy with FOLFIRINOX followed by chemoradiotherapy for locally advanced pancreatic cancer [54]. Unfortunately, VEGF inhibitors (VEGFi), largely used in many antineoplastic combination therapies for genitourinary and colon–rectal cancers, have considerable cardiovascular side effects, such as cardiomyopathy and arterial hypertension, which can affect up to one out of four patients treated with VEGFi [55, 56].

The role of natriuretic peptides in oncogenesis is also being evaluated [57, 58]. Intriguingly, circulating cardiovascular hormones, such as the N terminal pro peptide of the brain natriuretic peptide (NT-proBNP), have been shown to be related to cancer disease progression and severity, suggesting the presence of subclinical functional and morphological heart damage, providing hints for HF therapy in cancer patients beyond the focus of prevention of anticancer drug–induced cardiotoxicity [59].

Inflammation also plays a major role in the pathophysiology of both cancer and HF. Indeed, the role of inflammation in carcinogenesis has been extensively studied since the nineteenth century [60], and on the other hand, there is no doubt that a chronic inflammation state can be considered a cardiovascular risk factor [21]. For instance, the inflammatory state is involved in the initiation and progression of atherosclerotic processes [61••], ultimately leading to thrombosis, which underlies the development of ischemic heart disease [62••], and HF itself has been proven to be associated with increased circulating levels of pro-inflammatory cytokines [63, 64, 65•], leading to a state of mild chronic systemic inflammation [66]. Also, necrotic post-MI cells have an intense stress response, activating nuclear factor NF-kB [12•], one of the major tumor-promoting mechanism, which induces genes responsible for cell proliferation, survival, angiogenesis, and metastasis [67, 68•]. It has been demonstrated that clonal hematopoiesis of indeterminate potential (CHIP), defined as the presence of clonal leukocytes with impaired immune proprieties derived by acquired mutation in hematopoietic stem cells, may be associated with increase in CV events and all-cause mortality [69]. The risk of developing CHIP increases with aging, and thus it rarely results in development of hematologic malignancies; it seems to be tightly linked to increased CV events and worse HF prognosis [70, 71].

Interestingly, the Canakinumab Anti-Inflammatory Thrombosis Outcomes (CANTOS) trial showed that IL-1β-targeting antibody, canakinumab, not only was able to reduce the rate of recurrence of major adverse cardiovascular events in patients with previous myocardial infarction [72••] but also was able to lower the risk of incident lung cancer and its mortality rate [73••]. On the other hand, canakinumab did not reduce the incidence of other malignancies [73••]. Unfortunately, in the CANTOS trial, it was not possible to extensively assess the effects of canakinumab on non-small cell lung carcinoma (NSCLC) [73••]. NSCLC patients are being studied in the CANOPY trial, an on-going phase III case-control study that is evaluating canakinumab as a possible antineoplastic treatment for NSCLC in different settings, such as after adjuvant standard chemotherapeutical regiments (CANOPY-A), in association with pembrolizumab and platinum-derived compound (CANOPY-1) and in association with docetaxel (CANOPY-2) [74, 75].

Finally, interesting results from experimental work from Meijers and colleagues [76••] demonstrated that the presence of HF is indeed associated with enhanced tumor growth, independently from hemodynamic impairment, with involvement of cardiac-released factors [76••]. These results have also high translational value, since the authors observed increased plasma levels of their 5 candidate proteins in the plasma of 101 patients with chronic HF compared to 180 healthy patients enrolled in the PREVEND study [17, 77]. Also, increased HF biomarkers, as well as inflammation-related proteins, were predictive of incident cancer independent of cancer risk factors [78].

### Cancer in Heart Failure Patients: Clinical Implications

For clinicians, dealing with HF patients who develop cancer can be challenging, especially when facing possible symptomatologic overlaps between new-onset cancer and preexisting heart dysfunction (that can worsen the already poor outcome of HF patients) and when choosing the best combination of treatments for HF and cancer [1]. There are several clinical implications: new-onset cancer can impair the fragile homeostasis of the HF patients, can increase the risk of developing cardiotoxicity due to antineoplastic treatments, and can limit both oncological and cardiovascular therapeutic options, worsening the prognosis of this subset of patients. In addition, cancer itself can further impair cardiac function [79], by altering patients’ electrolyte homeostasis, producing hormonal alterations and worsening endothelial dysfunction and chronic inflammation [1].

Hasin and co-workers also demonstrated that patients with HF that develop cancer have a poorer prognosis compared to non-HF controls [14]. This is probably due to a higher prevalence of comorbidities in HF patients and the superimposed mortality risk derived by the coexistence of both cancer and HF [80••]. Cancer can also increase the risk of readmission for HF, further uprising the risk of death for those patients [81]. Banke and colleagues, comparing the incidence of cancer between HF patients and the general background population in Denmark, not only confirmed previous data showing that HF patients have a higher risk of developing new-onset cancer but also found that, after stratifying the population into 3 groups according to age, HF patients aged < 60 and 60–69 years old had the same mortality outcome compared to control patients in the groups aged 60–69 and > 70 years old, respectively [13•]. These data suggest that the mortality risk derived from cancer superimposed on the one derived for the preexisting HF, dramatically worsening patients’ prognosis. Furthermore, the symptoms of new-onset cancer may be masked by HF symptoms, and, also, the tumor can affect the already fragile homeostasis of patients with preexisting heart dysfunction [1, 79]. Additionally, the psychological burden must be addressed, considering that both HF and cancer could have a negative impact on the patient’s mental status, frequently leading to depression, a well-known cause of increased mortality rate in both diseases [82, 83].

Another important issue that needs to be addressed concerns antineoplastic treatment in HF patients. Patients with preexisting HF have a higher risk of developing cardiac toxicities induced by antineoplastic treatments, which can superimpose on an already dysfunctional heart [1, 6••, 7••, 8]. Additionally, patients with HF have a higher risk of mortality during anticancer surgery, compared to the non-HF population, which sometimes limits the antineoplastic treatment and affects prognosis [84•, 85].

It is fundamental for cardiologists and HF specialists to perform a comprehensive evaluation of HF patients who develop cancer, in order to fully assess their characteristics. A full baseline assessment is recommended, including clinical history, ECG, physical examination, blood withdrawal to exclude possible serum alteration (e.g., anemia, hyperglycemia), echocardiography, and any other test deemed necessary by the clinician. In this subset, it is essential to perform a risk–benefit analysis to truly understand what are the best possible treatments available for both cancer and HF and in order to tailor specific treatments for each patient, taking also into account cancer characteristics and HF stage and comorbidities [1, 86, 87••, 88••]. The first steps should be optimization of cardiac therapy and addressing the modifiable comorbidities (e.g., suggesting smoke cessation and diet adjustments, improving diabetes treatments) and, when necessary, treatment of underlying valvular defects or evaluation of residual ischemia [1, 4••]. Correction of metabolic disorders, electrolyte impairments, or any other coexisting alterations that could increase the risk of developing cardiotoxicity induced by antineoplastic treatments is also important [1, 6••, 7••, 8]. The process of optimization and up-titration of HF therapy may require even months; this sometimes can lead to a long time latency before starting antineoplastic treatments (e.g., surgery or chemotherapy) [1] and can impair patients’ survival. The interplay between cardiologists and oncologists is crucial in this phase, in order to guarantee the best management of the HF patients who develops cancer. It is fundamental to schedule regular follow-ups for HF–cancer patients in order to early identify any changes in HF symptoms that may be suggestive of new-onset cardiotoxicity, and promptly correct any serum alteration or other signs of decompensated HF. Withdrawal of cancer treatments should be avoided as much as possible to guarantee the completion of antineoplastic treatment to HF patient.

Fluid management during chemotherapy administration is another important issue. Cancer patients often are administered with large amount of fluids in order to limit renal adverse reaction induced by antineoplastic drugs [89••]. This approach is dangerous in HF patients, due to the risk of fluid overload, but, on the other hand, HF is often associated with chronic kidney disease, especially in older patients [90]. HF patients should be administered with lower doses of fluids, infusion time should be prolonged, and diuretics could be added/increased to avoid pulmonary edema or other complications related to volume overload [1, 4••].

Cancer patients also have an increased risk of developing QT prolongation and atrial fibrillation (AF) [91], which is a well-known cause of increased mortality risk in HF patients [92]. The possible causes behind new-onset AF in this subset seem to be thoracic surgery, electrolyte impairment, advanced age, metabolic alterations, and hypoxia [91]. Moreover, AF requires anticoagulation treatment, which may be contraindicated in HF patients with new-onset cancers, due to the high risk of bleeding (i.e., gastrointestinal malignancies and brain metastases have high bleeding risk [93]). It is also important to periodically interrogate patients’ electronic devices, such as implantable defibrillators and pacemakers, with special attention to radiation therapies that can impair device functionality [94•].

Bottomline, it is fundamental for HF patients to be administered the best HF treatments in order to be able to receive the most suitable anticancer therapies: HF should not burden antineoplastic management. Indeed, Gross and colleagues highlighted that patients with HF and colon–rectal cancer are less likely to receive adjuvant chemotherapy and have a worse 5-year survival outcome when cancer remained untreated, compared to non-HF patients with cancer [95]. On the other hand, another critical issue to be addressed is the risk of undertreating HF after cancer diagnosis. HF patients usually are administered with a complex therapy and also with other drugs to treat comorbidities, such as diabetes, thyroid dysfunction, and dyslipidemia [4••]. However, the presence of cancer can alter HF patients’ homeostasis, leading to temporary changes in HF therapy, with possible reduction and suboptimization. Patients with HF and cancer can manifest vomiting, diarrhea, or other endocrinological conditions that may affect electrolyte status, forcing cardiologists to reduce important HF treatment medications.

Another important problem is how to manage anticoagulation therapy in HF patients with cancer. It is well known that cancer may have both a pro-thrombotic and a pro-hemorrhagic effect, depending on the cancer type and its site (e.g., patients with gastrointestinal cancer have a higher risk of major bleeding, especially when administered with anticoagulants [93]). As stated above, AF is a common comorbidity in both HF and cancer patients, and it is also a possible side effect of some anticancer treatments [91, 92, 94•]. In recent years, direct oral anticoagulants are being preferred to warfarin thanks to their safety profile [96]. Moreover, HF–cancer patients have a higher risk of developing deep venous thrombosis and pulmonary embolisms, as well as central venous catheter thrombosis [96]. For this reason, it is fundamental for the cardio-oncology team to stratify the thrombosis/bleeding risk for each HF–cancer patient and decide whether anticoagulation treatment is required and which anticoagulants are to be chosen among low-weight heparin and direct oral anticoagulants, considering among all possible drug interactions [97••].

Of notice, patients with active cancer are almost automatically excluded from the heart transplant list, while a careful dialog between cardiologists and oncologists is recommended [98] in order to identify whether the patient is suitable for heart transplantation, identifying life expectancy and prognosis [1, 4••]. Many other invasive treatments for HF are being investigated in the presence of a concomitant cancer [99] diagnosis that shortens patients’ life expectancy. For example, device implantation is vetoed when life expectancy is shorter than 1 year [4••]. On the other hand, treatment with left ventricular assist devices should be considered in patients with concomitant cancer who have a good prognosis (≥ 2 years) [4••, 100]. However, when considering patients for cardiac transplant or left ventricular devices, it is fundamental to identify the prognosis and the life expectancy, taking also into account cancer types and its specific prognosis. Furthermore, HF patients who undergo heart transplantation will have to be administered with immunosuppressant therapy for life, and this is a well-known risk factor for cancer development. Whether patients with recent malignancies are more exposed to the risk of developing a new tumor with immunosuppressive therapies is still a matter of debate. However, it is clear how every patient with preexisting HF who develops cancer must be treated as a unique case and needs a medical approach tailored to this peculiar condition. Also, clinicians should risk-stratify HF patients in order to identify those who have higher risk of developing cancer and identify screening protocols in order to anticipate cancer diagnosis.

Finally, dexrazoxane may have an important cardioprotective role even in HF patients with new-onset cancer. Dexrazoxane is well known for its cardioprotective effects and has been largely used to treat anthracycline-induced cardiotoxicity [101, 102]. However, there are no studies that evaluate the efficacy and safety of dexrazoxane in HF patients, and, additionally, its cardioprotective effects are limited to anthracyclines, which are still pivotal in many antineoplastic treatments. Indeed, there is urgent need for researching other cardioprotective agents which may counteract and prevent cardiotoxicity induced by drugs other than anthracyclines.

## Conclusions

Recent data show that not only the connection between cancer and HF is stochastic but also these two diseases are deeply linked by common pathophysiological pathways, and the presence of preexisting HF is per se a risk factor for cancer development. The risk of developing cancer in HF patients is higher than in the general population, and management of these patients is burdened by the complexity of these two diseases and their interactions. It may be recommended to consider differential surveillance programs to screen patients with HF who are at risk for cancer development to anticipate the diagnosis of possible new-onset malignancies. However, further studies on cardiac-secreted factors [78] and their potential utility as cancer biomarkers could help to risk-stratify patients with HF in terms of their cancer risk.

Clinicians have to face that cancer and HF carry an independent risk of mortality, but also potentially hinder the treatment of one another, with the result being a further increase in mortality, not to speak of the well-known HF risk related to treatment with anticancer drugs that may even worsen the scenario of cancer superimposed to preexisting HF [1]. Moreover, the lack of international guidelines on the correct management of patients affected simultaneously by cancer and HF should be addressed.

In order to overcome these challenges, a close collaboration between cardiologists and oncologists is fundamental to improve the management of these patients, with both specialists understanding the benefits of therapy for HF and cancer, and the risks of withholding or sub-optimally treating either or both diseases. Cardiologists and oncologists could collaborate by creating national or regional cardiotoxicity registries to better evaluate the incidence of cardiac adverse reactions due to antineoplastic treatments and explore the possible presence of other CV side effects still unknown or underdiagnosed.

The prognostic impact of each condition should always be well defined and considered in decision-making [1]. A multidisciplinary approach is encouraged [98] and should include other healthcare professionals, including cardiac rehabilitation, psychology, and palliative care where necessary.

The scientific evidence upon which clinical decisions can be based is very limited, but epidemiology is showing that occurrence of cancer in HF is an increasingly common problem with an aging population and in the current era of cancer and post-myocardial infarction survivorship. While the SCHOLAR [103] and SAFE-Heart [104••, 105] studies addressed congestive HF that occurred with anthracycline/anti-HER2 treatments as opposed to preexisting HF, they were able to show that most of their cohorts could be medically managed with medical therapy to continue and complete their breast cancer treatments. Likewise, this approach could apply to other populations with preexisting HF with cancer, but would need further data. Further well-designed studies are required to clarify the thresholds at which cancer treatment should not be administered to patients with preexisting HF and the optimal cardioprotective and surveillance strategies for patients in whom these two worrisome conditions coexist. Clinical trials should be conducted specifically on HF patients who develop cancer, in order to better understand and define clinical course, antineoplastic and HF drug dosages, and possible additional treatment-related side effects that those patients may face. Moreover, more trials testing anticancer treatment should include a real-world population, which also include patients with many CV comorbidities to improve current knowledge on the impact that cancer has on preexisting CVDs.

Finally, more attention should be given to prevention of the development of cancer in HF patients, acting on education and information to reduce modifiable common risk factors between cancer and HF (i.e., anti-smoking campaigns, obesity prevention…). Table 1 summarizes the key points discussed above.

**Table 1:** Summary of the key points of this review on cancer risk in heart failure

Key points	
• In Western countries, cancer and heart failure (HF) are two of the most common diseases in an aging population and are responsible for most of the deaths. • These two conditions share numerous risk factors, such as obesity, smoking, and hyperglycemia. Furthermore, the link between HF and cancer involves many molecular pathways that characterize HF, suggesting that the presence of HF per se might be a risk factor for developing cancer. • It has been demonstrated that patients with HF have a higher incidence of cancer compared to the general population. Moreover, patients with HF and cancer have a poorer prognosis. • The management of HF patients who develop cancer is complex and requires a constant interplay between cardiologists and oncologists.	
